# Revisiting the Effects of Xenon on Urate Oxidase and Tissue Plasminogen Activator: No Evidence for Inhibition by Noble Gases

**DOI:** 10.3389/fmolb.2020.574477

**Published:** 2020-09-11

**Authors:** Jesse Cahill, Anne M. Ruffing

**Affiliations:** Sandia National Laboratories, Molecular and Microbiology Department, Albuquerque, NM, United States

**Keywords:** xenon, urate oxidase, tissue plasminogen activator, noble gas-protein interaction, protein sparging, protein denaturation, liquid-gas interface

## Abstract

Although chemically inert, Xe and other noble gases have been shown to have functional effects on biological systems. For example, Xe is a powerful anesthetic with neuroprotective properties. Recent reports have claimed that Xe inhibits the activity of tissue plasminogen activator (tPA) and urate oxidase (UOX), indicating that the use of Xe as an anesthetic may have undesirable side effects. Here, we revisited the methods used to demonstrate Xe inhibition of UOX and tPA, testing both indirect and direct gas delivery methods with variable bubble sizes and gas flowrates. Our results indicate that Xe or Kr do not affect the activity of UOX or tPA and that the previously reported inhibition is due to protein damage attendant to directly bubbling gases into protein solutions. The lack of evidence to support Xe inhibition of UOX or tPA alleviates concerns regarding possible side effects for the clinical application of Xe as an anesthetic. Furthermore, this study illustrates the importance of using indirect methods of gas dissolution for studying gas-protein interactions in aqueous solution.

## Introduction

Noble gases have been shown to have functional interactions with biological systems (Winkler et al., 2016). As of this report, there are hundreds of structures in the protein database that are complexed with noble gases (Xe, Kr, and Ar). Crystallography has shown that the binding of noble gases to hydrophobic cavities is correlated with their size and polarizability (Xe > Kr > Ar) (Colloc’h et al., 2011). The biological effects and water solubility of noble gases are similarly correlated with increasing molecular weight, i.e., Xe > Kr > Ar.

The most well-described protein-noble gas interaction is with Xe, in part because ^129^Xe is used as a probe for NMR spectroscopy. The affinity of ^129^Xe for small hydrophobic cavities and chemical shift sensitivity have been shown to report on protein conformational changes (Locci et al., 2001; Lowery et al., 2004, 2005).

Perhaps the most impactful biological effect of noble gases is their potential for use in medicine. Xe, and to a lesser extent Kr and Ar, have anesthetic and neuroprotective properties (Winkler et al., 2016). However, the use of Xe in clinical settings is precluded by its scarcity (Neice and Zornow, 2016) and the lack of fundamental understanding of the mechanisms of Xe-protein interaction. Recent studies have identified other Xe-protein interactions that could complicate its use in some settings. Xe is reported to inhibit tissue-type plasminogen activator (tPA), a serine protease approved to treat ischemic stroke (David et al., 2010). Additionally, Xe was shown to cause inhibition of urate oxidase (UOX) upon exposure to a 75% Xe/25% O_2_ mixture (Marassio et al., 2011). UOX has been used in clinical settings to treat gout by reducing uric acid levels in the blood (Bomalaski et al., 2002; Pui, 2002; Yang et al., 2012). Although the likelihood that Xe will be administered with these therapeutics is small, these studies highlight unexpected and potentially significant off-target effects for using Xe in medicine.

Importantly, structural data for UOX or tPA interaction with noble gases are obtained under hyperbaric conditions (Colloc’h et al., 2011; Marassio et al., 2011). In contrast, enzyme activity assays are performed by sparging (bubbling) solutions with gases. As such, there is no structural information supporting whether noble gas-protein complexes are formed when noble gases are delivered by sparging under atmospheric conditions that are relevant for clinical applications. The method used to expose protein to gas is an important consideration since the direct delivery of gases to protein solutions causes protein damage (Caussette et al., 1998, 1999; Thomas and Geer, 2011; Xiao and Konermann, 2015). Direct sparging was reported as the gas delivery method in the study claiming Xe inhibition of tPA (David et al., 2010), and this procedure was cited by the report on Xe inhibition of UOX (Colloc’h et al., 2011). Therefore, we suspected that some of the reported Xe inhibition may be due to protein damage rather than specific complex formation between Xe and UOX or tPA. In this study, we revisit whether noble gases affect UOX and tPA activity, comparing the effects of a direct sparging method to an indirect method of mixing the enzymes with sparged buffer solutions.

## Materials and Methods

### Materials

All gases used in this study (80% N_2_/20% O_2_, 80% Kr/20% O_2_, 80% Xe/20% O_2_, Ar, Kr, and Xe) were provided by Matheson Tri-Gas, Inc. *Escherichia coli* strains DH5α and BL21(DE3) were provided by NEB and ATCC, respectively. 8-azaxanthine (98%, HPLC-grade) was purchased from Sigma-Aldrich. BugBuster was purchased from Novagen. The remaining chemicals used in this study were manufactured by Fisher Bioreagents (Luria Broth, NaCl, Tris-Cl, IPTG) and Fisher Scientific (all other chemicals).

### Construction of UOX Overexpression Vector

A His-UOX overexpression vector was prepared by GenScript by subcloning a codon-optimized *Aspergillus flavis* urate oxidase gene into the pET-11a vector under control of the T7 promoter using the *Xba*I and *Bam*HI sites (Supplementary Figure S1). This vector, denoted pHis-UOX, contains the a 10x His tag and linker region GHHHHHHHHHH-SSGHIDDDDKHM, inserted after the start codon.

### Protein Overexpression

pHis-UOX was transformed into *Escherichia coli* BL21(DE3) and selected on LB agar plates supplemented with 100 μg/mL ampicillin and 0.2% glucose. Two colonies were inoculated for overnight aerobic growth each in 3 mL of LB-amp-glucose media. The next day, a subculture was performed (1:100) into 1 L of LB-amp media, split into four 1 L baffled flasks and grown at 37°C and 250 rpm for 2.5 h in a New Brunswick Innova 42R shaking incubator. At A_600_ ∼0.6, cultures were induced with 1 mM of IPTG (final concentration) and grown overnight at 18°C and 250 rpm for 18 h. Cultures were centrifuged for 25 min at 5000 × g and 4°C using a Sorvall Evolution RC Superspeed centrifuge. The cell pellet was resuspended in 10 mL of 100 mM Tris buffer pH 8.2 with 100 mM NaCl and stored at −80°C until purification.

Cell pellets were thawed on ice and lysed using BugBuster reagent in the presence of Halt protease inhibitor cocktail (Thermo Fisher, Waltham, MA), both used according to manufacturers’ instructions. The lysate was dialyzed using 20 kDa MWCO Slide-A-Lyzer (Thermo Fisher) cassettes against 100 mM Tris-Cl pH 8.2 with 100 mM NaCl and His-purified using a HisPur Ni-NTA Spin Purification kit (Thermo Fisher) according to manufacturer’s instructions, except the pH of the buffers used in the His-purification were adjusted to 8.3 to maintain protein stability. Additionally, we used a fourth wash step in 3x imidazole and collected a fourth elution in a half-volume (1.5 mL) of the elution buffer. Elutions 1–4 were combined and dialyzed again into 100 mM Tris-Cl pH 8.2 with 100 mM NaCl for *in vitro* experiments measuring UOX activity. The concentration of purified His-UOX was 1.84 mg/mL, determined using Coomassie Plus Bradford Protein Assay (Thermo Fisher Scientific). Aliquots were saved at −80°C.

### Gas Delivery

#### Sparging Using a Blunt Needle

Prior to sparging, the gas line was purged for 10 min to remove air from the line. Vented conical tubes were prepared by drilling two 3/32-inch holes into the top of 20 mm butyl stoppers (Chemglass Life Sciences). One hole was used for delivery of gas through a 14-gauge, 6-inch blunt end needle (Cadence Science), and the venting hole was supported by an 18-gauge blunt end needle. Stoppers were used with aluminum crimp caps (Chemglass Life Sciences) to make a seal, and the needle was positioned just above the bottom of the conical tube. Likewise, 1.5 mL microcentrifuge tubes were prepared for gas delivery by drilling two 3/32 inch holes in the caps. We used the 1–2 mL of reaction buffer with (direct) or without (indirect) His-UOX in a conical tube for cuvette kinetic measurements made on the NanoDrop One and Jasco Circular Dichroism Spectrophotometer. For kinetic measurements on a microtiter plate, gases were bubbled into 300 μL of reaction buffer with or without enzyme (His-UOX or tPA) in closed tubes using an 18-gauge blunt end needle. Flow rate was controlled by a Matheson rotameter 7300 with 610A flow tube and an upstream pressure of 5 psi, corresponding to a flow rate of 65–70 mL/min.

#### Sparging Using a Glass Dispersion Tube

Prior to sparging, the gas line was purged for 10 min to remove air from the line. Gas was delivered through a glass dispersion tube (Ace glass, porosity 4–8 μm) into 3 mL of reaction buffer (indirect) or 3 mL of enzyme (His-UOX or tPA) in reaction buffer (direct) in a 15 mL conical tube for 5 min. See the kinetic measurements descriptions for compositions of the reaction buffers. Flow rate corresponded to 12–15 mL/min.

### Kinetic Measurements for UOX

#### Urate Disappearance Method

Urate disappearance was monitored from absorbance at 293 nm on a Thermo Scientific NanoDrop One UV-Vis Spectrophotometer. We added 928 μL of reaction buffer (100 mM Tris-Cl, pH 8.2 with 100 mM NaCl), which was treated or untreated with gas as described above to 50 μL of urate solution (100 μM final volume) in a quartz cuvette. 22 μL of His-UOX (1 μM final concentration) was immediately added to the cuvette which was sealed, and contents were mixed by three inversions. Absorbance measurements were taken immediately after the third inversion and monitored every 10 s at 25°C for 15 min. Readings were blanked to substrate-free reaction mixtures and initial velocities were obtained from datapoints taken during first 90 s. For experiments using competitive inhibitor 8-azaxanthine (8-AZA), the urate solution was supplemented with equimolar concentrations of 8-AZA. Each datapoint is the average of three independently-sparged replicates, and the unsparged control measurements were collected at the start, middle, and end of each batch to verify enzyme stability during each experimental run.

#### Amplex Red Method

UOX kinetic assays were performed using the Amplex Red Uricase Assay kit (Thermo Fisher Scientific) according to the manufacturer’s instructions. Experiments were conducted similarly to the urate disappearance method except 267 μL of reaction buffer was added to 30 μL of His-UOX (1 μM final concentration), 2 μL Amplex Red and 0.8 μL horseradish peroxidase (HRP). Reactions were monitored in a Corning 3631 microtiter plate (black, clear bottom, no lid) and read immediately using a BioTek Cytation 5 plate reader pre-heated to 37°C. Measurements were made every 2 s for 3 min using a Xenon flash light source and monochromator detector. Excitation and emission settings were 530/20 and 590/30, respectively. Initial velocities were obtained from the first 18 s (linear range) of data. Each datapoint is the average of three independently-sparged replicates, and the unsparged control measurements were collected at the start, middle, and end of each batch to verify enzyme stability during each experimental run.

### Kinetic Measurements for tPA

Human recombinant tPA (P1324-100) and its inhibitor, human recombinant plasminogen activator inhibitor-1 (PAI-1) (6377-100) were purchased from Biovision, Inc. tPA was reconstituted in tPA reaction buffer (30 mM Tris-HCl, pH 8.4, 100 mM NaCl) at a concentration of 235 μg/mL or 4 μM, 10x the final assay concentration reported previously for measuring noble gas inhibition of tPA (David et al., 2010). Concentrated tPA aliquots were stored at −20°C and thawed to room temperature just prior to the kinetic measurement. Chromogenic tPA substrate, CH_3_SO_2_-D-HHT-GLy-Arg-pNA∙AcOH (T2943, Sigma-Aldrich, Inc.), was dissolved in ultrapure water to 10x stock concentrations of 100 μM, 1 mM, 5 mM, and 10 mM. Chromogenic tPA substrate stocks were stored at 4°C and warmed to room temperature just prior to the kinetic measurement. For tPA kinetic measurements, 10 μL of chromogenic tPA substrate was added to a Corning 3631 black with clear-bottom 96-well microplate. The 10x tPA stock was mixed with unsparged or gas-sparged reaction buffer in a 1.5 mL tube, and 90 μL of the tPA mixture was immediately added to the well containing the chromogenic substrate, such that the final concentration of tPA was 0.4 μM. Absorbance at 405 nm was measured at 25°C every 2 s for 5 min using a BioTek Cytation 5 reader. The initial velocity was calculated from the linear slope at the start of the reaction, which varied from the first 12–40 datapoints depending on the substrate concentration (10 μM–1 mM). Each datapoint is the average of three independently-sparged replicates, and the unsparged control measurements were collected at the start, middle, and end of each batch to verify enzyme stability during each experimental run.

### Circular Dichroism (CD) Measurements

CD spectra were obtained using a Jasco J-815 spectropolarimeter. His-UOX was diluted to 10 μM in UOX reaction buffer and dialyzed extensively in 10 mM sodium phosphate pH 8.0. His-UOX (1 μM final) was mixed with Xe/O_2_-sparged phosphate buffer, sparged directly with Xe/O_2_ as described above, or unsparged, and 1400 μL of this mixture was loaded into a 0.1 cm quartz cuvette (Starna Cells, Inc.) and immediately sealed. Temperature in the cell was maintained at 22.0°C by a Jasco PFD-425S/15 Peltier device. Eight scans from 260 to 190 nm were performed at 20 nm/min with a 0.5 nm step size. Each condition above was measured three independent times including buffer-only baseline scans. Scans were accumulated, averaged, and subtracted from the average baseline.

## Results

### No Change in UOX Activity With Indirect Gas Delivery

**FIGURE 1:**
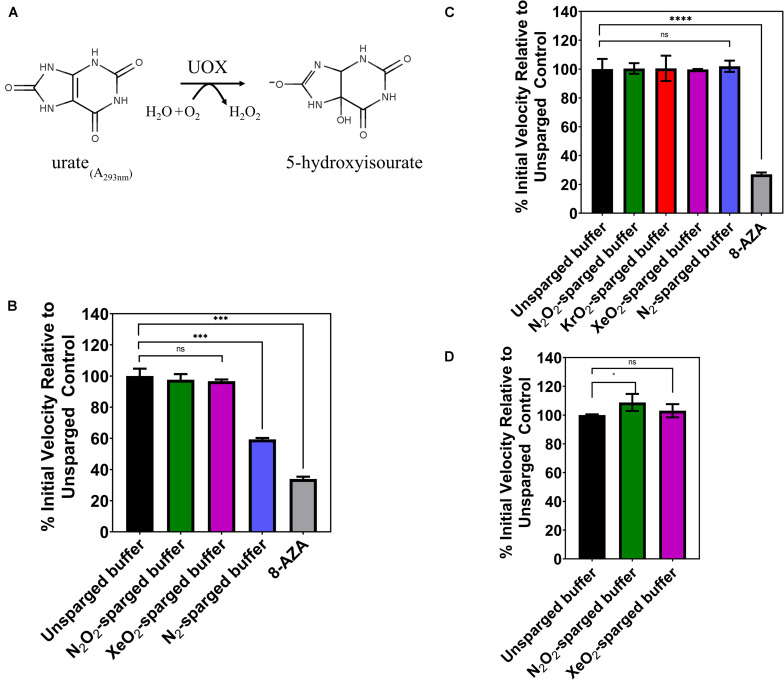
**(A)** UOX reaction: UOX degrades urate into 5-hydoxyisourate. Activity can be measured by monitoring disappearance of urate at 293 nm **(B)** or measuring hydrogen peroxide production. **(C,D)** Gases were delivered by a blunt-end needle **(A,D)** or a glass dispersion tube **(B,C)**. The reaction buffer was sparged with the indicated gas and was immediately mixed with urate and His-UOX in a sealed cuvette and monitored on a UV-Vis spectrophotometer **(A)**. For **(B,D)**, the sparged reaction buffer was mixed with His-UOX (1 μM final) and added to urate (100 μM final) solution in a microtiter plate. The percent activity is calculated from the initial velocity of each set divided by the average initial velocity of the unsparged set multiplied by 100%. 8-AZA, UOx inhibitor 8-azaxanthine, used at 1:1 stoichiometric ratio to urate. All gases contain 20% oxygen except N_2_, which is pure nitrogen. Error bars correspond to standard deviation for three or more independently sparged sets. Significance determined by *t*-test and denoted by the following: ^*ns*^*P* > 0.05, *P ≤ 0.05, **P ≤ 0.01, ***P ≤ 0.001, ****P ≤ 0.001.

In 2011, Marassio et al. (2011), presented evidence that Xe inhibits the activity of *Aspergillus flavus* UOX *in vitro*. Structural analyses of UOX crystals exposed to pressurized Xe supported the authors’ interpretation that the mechanism of inhibition was the expansion of the gas binding site when occupied by Xe (Marassio et al., 2011). To further study this effect, we attempted to recapitulate the kinetic analysis using the same concentrations of substrate, enzyme, and detection method but employing an indirect gas delivery method to prevent physical denaturation of the enzyme and to determine the chemical effect of noble gases on UOX activity. We sparged the reaction buffer with 80/20 mixtures of N_2_/O_2_, Xe/O_2_ and pure N_2_ gas using a blunt-end needle to deliver gas into a vented conical tube. Gas-treated buffers were mixed with 100 μM urate and 1 μM His-UOX in a sealed cuvette, and initial velocities were obtained by measuring the rate of urate disappearance at 293 nm (Figure 1A). As expected, equimolar concentrations of urate and 8-AZA caused significant inhibition of UOX activity (Figure 1B). Since oxygen is required for UOX activity, we sparged the reaction buffer with pure nitrogen, which would be expected to displace oxygen from the solution. This control showed a > 40% decrease in UOX activity, indicating that indirect sparging was effective for gas delivery. However, contrary to the previous study, we detected no significant difference in activity for His-UOX in reaction buffer treated with Xe/O_2_ (Figure 1B). During UOX catalysis, hydrogen peroxide production occurs stoichiometrically along with the formation of 5-hydroxyisourate. Measurements of UOX activity based on production of hydrogen peroxide have been shown to have a higher confidence interval than the urate disappearance method (Fraisse et al., 2002). Therefore, we used a commercial Amplex Red Uricase Assay kit, which reacts with hydrogen peroxide and HRP to convert Amplex Red to resorufin, a red fluorophore. However, as before, treatment of the reaction buffer with N_2_/O_2_, Kr/O_2_, or Xe/O_2_ did not lead to a detectable difference in UOX activity (Figure 1C). In some studies, Kr is reported to have similar biological effects as Xe (Kennedy et al., 1992; Colloc’h et al., 2011). However, we detected no significant difference for UOX activity in Kr/O_2_-treated buffer. Additionally, exposure of His-UOX to reaction buffer treated with pure nitrogen did not result in a statistically significant change in UOX activity (Figure 1C), likely due to the production of molecular oxygen by HRP. A blunt-end needle was also used to sparge the reaction buffer prior to mixing with His-UOX. In contrast to previous data, there was a slight increase in UOX activity exposed to N_2_/O_2_ and Xe/O_2_-treated reaction buffers but this was only significant in the case of N_2_/O_2_ (Figure 1D). The simplest explanation for the increase in activity was that this treatment increased amount of molecular oxygen solubilized in the smaller volume of reaction buffer, which increased the availability of molecular oxygen for His-UOX catalysis.

### No Change in tPA Activity With Indirect Gas Delivery

**FIGURE 2:**
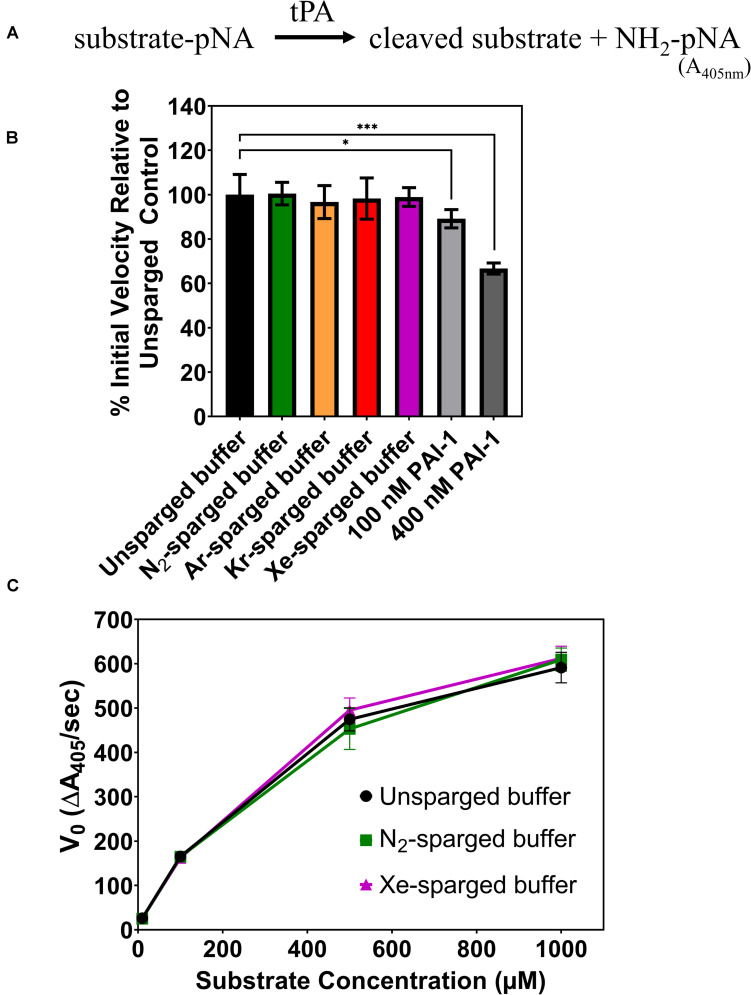
**(A)** Schematic of the tPA assay. Chromophore p-nitroanilide is released as tPA hydrolyzes the substrate. **(B)** Percent of tPA activity in sparged buffer solutions relative to the unsparged control with a substrate concentration of 100 μM. Two concentrations of a standard tPA inhibitor (PAI-1) are included as controls. **(C)** Initial velocity of tPA activity in sparged buffer solutions across a range of substrate concentrations. In all panels, tPA activity is measured as the initial velocity, which is the linear slope at the start of the reaction. All measurements are averages of 3 independently sparged biological replicates with error bars indicating the standard deviation. Significance determined by *t*-test and denoted by the following: ^*ns*^*P* > 0.05, *P ≤ 0.05, **P ≤ 0.01, ***P ≤ 0.001, ****P ≤ 0.001.

In 2010, David et al. (2010), reported that Xe inhibited the catalytic efficiency of tPA in aqueous solution by as much as 80%. Again, we sought to verify these results by investigating the effects of noble gases on tPA activity using an indirect sparging method and a commercial recombinant human tPA with an amino acid sequence identical to the Actilyse tPA used in previous studies (David et al., 2013). The tPA assay measures colorimetric change resulting from tPA hydrolysis of a synthetic substrate, which releases a p-nitroanilide chromophore (Figure 2A). Like the UOX work above, gases were delivered through a gas dispersion tube into tPA reaction buffer prior to the assay. The sparged reaction buffer was immediately mixed with concentrated tPA and added to the substrate with final tPA and substrate concentrations of 0.4 and 100 μM, respectively. There was no significant change in tPA activity for any of the gases tested (N_2_, Ar, Kr, Xe) (Figure 2B), whereas the standard inhibitor, plasminogen activator inhibitor-1 (PAI-1), showed a dose dependent inhibition of tPA activity. As the effect of enzyme inhibitors can vary across substrate concentrations, we also tested tPA activity across substrate concentrations ranging from 10 μM to 1 mM with unsparged, N_2_-sparged, and Xe-sparged buffers while keeping the tPA concentration at 0.4 μM, similar to the previously published studies (David et al., 2010, 2013). There was no significant change in tPA activity for any treatment across all substrate concentrations (Figure 2C). We also tested indirect gas delivery using a blunt needle to mimic the gas delivery system reported in David et al. (2010), and again, there was no significant change in tPA activity with noble gas treatment (Supplementary Figure S2).

### Direct Sparging of UOX or tPA Reduces Enzyme Activity Measurements

**FIGURE 3:**
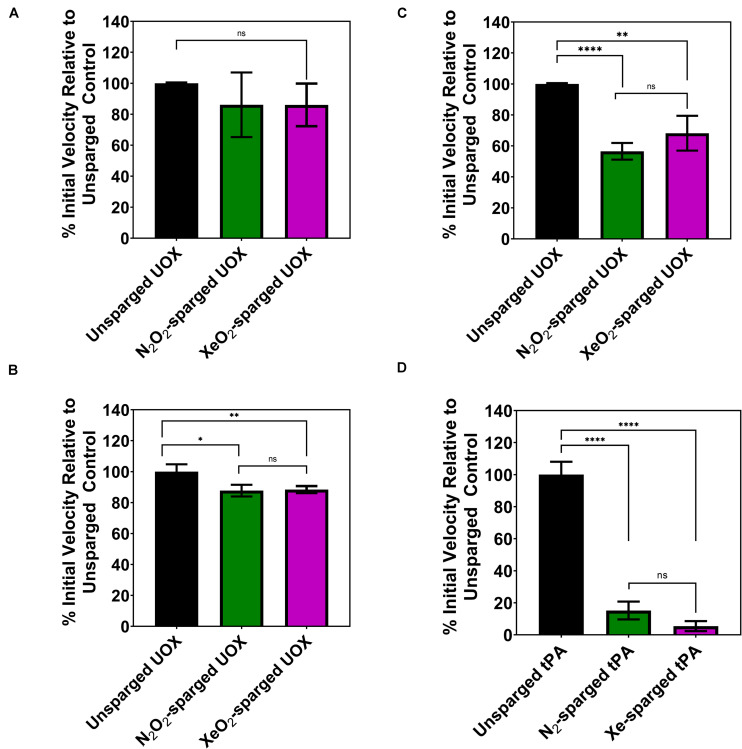
Activity of His-UOX and tPA after direct sparging of the enzyme with a glass dispersion tube **(A)** or blunt-end needle **(B–D)**. Percent activity of UOX measured by peroxide accumulation method **(A,C)** and the urate disappearance method **(B)**. For UOX and tPA, direct sparging sets **(A–D)** above treated identically to Figures 1B–D, 2B (respectively) except that enzyme solutions were directly sparged with the indicated gas prior to mixing with substrate. **(D)** Percent activity of tPA with direct sparging of the enzyme with N_2_ or Xe. Error bars correspond to standard deviation for three or more independently sparged sets. For UOX, all gases contain 20% oxygen. Significance determined by *t*-test and denoted by the following: ^*ns*^*P* > 0.05, *P ≤ 0.05, **P ≤ 0.01, ***P ≤ 0.001, ****P ≤ 0.001.

As the indirect gas delivery methods did not show reduced UOX or tPA activity with noble gases, we tested whether direct sparging of enzyme solutions affects the activity measurements. Due to the large amount of enzyme required for direct sparging with the gas dispersion tubes, this gas delivery method was only tested with UOX. As expected, direct His-UOX sparging with the gas dispersion tube resulted a copious amount of foam (Supplementary Figure S3), indicative of protein damage (Clarkson et al., 1999). Surprisingly, the treatment did not result in a significant change in the average inhibition of His-UOX; however, direct sparging increased the variability between independently sparged sets (Figure 3A). To directly mimic the gas delivery used in previous studies (David et al., 2010; Marassio et al., 2011), we used a blunt-end needle to deliver gases at a higher flow rate into a 2 mL His-UOX solution in a vented conical and a 300 μL volume of His-UOX solution in a vented microcentrifuge tube (Supplementary Figures S4A,B). Direct sparging of His-UOX solutions with N_2_/O_2_ and Xe/O_2_ caused a ∼12% reduction of UOX activity in the conical (Figure 3B) and 44 and 32% (respectively) reduction in activity when delivered into a smaller volume in the microcentrifuge tube (Figure 3C). Similarly, direct sparging of tPA solutions using a blunt-end needle resulted in a 85–95% reduction in tPA activity, independent of the chemical composition of the gas (Figure 3D).

### Direct Sparging of UOX Reduces the CD Spectra

**FIGURE 4:**
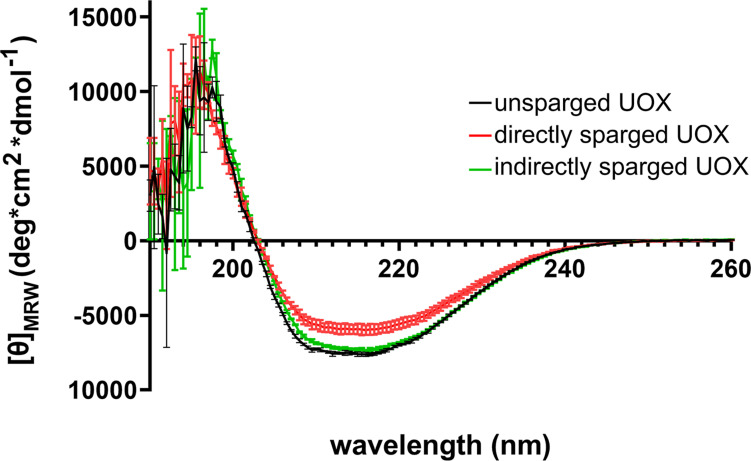
CD spectra of 1 μM His-UOX. Spectra obtained from His-UOX without sparging, mixed with Xe/O_2_− sparged phosphate buffer (9 parts sparged buffer to 1 part His-UOX), or directly sparged with Xe/O_2_. The average of three independent replicates is shown and error bars correspond to standard deviation.

Numerous studies have shown that introducing gas-liquid interfaces by the direct sparging of protein solutions results in reduced protein activity due to aggregation and denaturation (Caussette et al., 1998, 1999; Thomas and Geer, 2011; Xiao and Konermann, 2015). Such effects may result in changes to secondary structure that would be detectable by CD. Therefore, we compared the CD spectra of His-UOX exposed to Xe/O_2_-sparged buffer as well as His-UOX directly sparged with Xe/O_2_. The spectra shifted upward for the directly sparged sample, but the shape of the spectra did not change (Figure 4). The simplest interpretation is that the direct sparging treatment produced insoluble His-UOX aggregates that were not detectable by CD measurement, resulting in reduction in the absolute signal.

## Discussion

Structures of protein-noble gas complexes are formed by exposing crystallized protein to hyperbaric gas (Schoenborn et al., 1965; Locci et al., 2001). This raises the question of whether protein-noble gas interaction occurs at atmospheric pressures or conditions that would be relevant to clinical applications. Very few biochemical studies have investigated this question using purified proteins; however, Xe was reported to inhibit UOX and tPA activity *in vitro* when Xe was introduced by sparging (David et al., 2010; Marassio et al., 2011). There is no method to quantitatively compare the mass transfer of pressurized noble gases with protein crystal matrices to the infusion of aqueous enzyme solutions with noble gases by sparging. However, we can estimate the amount of Xe solubilized in the buffer used in our assays. At 1 atm, the mole fraction is 7.89 × 10^–5^ in 25°C water (Gevantman, 2000), which corresponds to 4.3 mM Xe in water. For the UOX assays in Figure 1B, the reaction mixture was kept in a sealed cuvette to minimize release of Xe from solution. These assays had the smallest dilution (7%) of the sparged buffer due to the addition of UOX and substrate. The components in our reaction buffer – Tris-Cl and NaCl -would be expected to reduce the solubility of Xe. There is a 9% reduction in the Ostwald solubility coefficient for Xe in 0.9% NaCl compared to pure water at 25°C (Yeh and Peterson, 1964). Therefore, a conservative estimate of the concentration of Xe after sparging with 80/20 Xe/O_2_ would be in the range of 1–3 mM. This suggests that in our assays there is a Xe:enzyme ratio of > 1000:1.

Importantly, the methods used to introduce Xe to UOX were unclear in the previous report (Marassio et al., 2011). In the case of tPA, Xe was directly sparged into tPA solutions (David et al., 2010, 2013). Furthermore, the corresponding author was unable to confirm the method of gas delivery for UOX (personal communication). The gas delivery method is critical since it has been well-established that introducing gas-liquid interfaces by the direct sparging of protein solutions results in reduced protein activity due to aggregation and denaturation (Caussette et al., 1998, 1999; Thomas and Geer, 2011; Xiao and Konermann, 2015). Therefore, it was uncertain whether the reported inhibition was due to specific Xe-protein contacts, as indicated by structural data, or caused by protein damage resulting from direct sparging. In this report, we attempted to recapitulate Xe inhibition of UOX or tPA and address this question by comparing the effects of exposing concentrated tPA or His-UOX to gas treated buffers versus directly sparging the enzymes. We detected no significant inhibition of activity when His-UOX or tPA were mixed with sparged buffers, except the expected reduction in UOX activity when its substrate oxygen was displaced by sparging with pure nitrogen (Figure 1B).

We confirmed that direct sparging results in a significant decrease in enzyme activity measurements (Figure 3). The reduction in measured activity is independent of the chemical composition of the gas, which indicates that this effect is non-specific and likely caused by physical protein damage. The upward shift of the CD spectra detected for directly sparged His-UOX has been observed in other studies using CD to measure protein integrity after exposure of enzymes to air-liquid interfaces (Cao and Tan, 2004; Duerkop et al., 2018). The shift for directly-sparged His-UOX is consistent with the formation of insoluble His-UOX aggregates caused by the introduction of air-liquid interfaces. For His-UOX, we demonstrate that the direct sparging treatment is dose dependent. That is, fine bubbles delivered by a gas dispersion tube at a low flow rate does not significantly reduce UOX activity. In contrast, a more aggressive sparging method using a blunt-end needle (David et al., 2010) resulted in up to a 43% decrease in UOX activity and up to a 95% decrease in tPA activity. Taken together, we cannot attribute the reduction of UOX or tPA activity to the protein-gas contacts reported in structural studies.

Given the rising interest in protein-noble gas interaction, it is likely that there will be an increase in biochemical analyses designed to probe the effects of noble gases on biological systems. While structural data and predictive software have shown that noble gases have unexpected interactions with diverse groups of proteins (Winkler et al., 2016, 2019), it will be important to use carefully designed *in vitro* studies to investigate whether these interactions occur under physiologically relevant conditions. The results of this work highlight an unreported methodological consideration that should be accounted for in future biochemical investigations of noble gas-protein interactions. Directly sparging protein solutions with gases results in protein damage, independent of the chemical composition of the gas.

## Data Availability Statement

All datasets generated for this study are included in the article/Supplementary Material.

## Author Contributions

JC and AR contributed to conception and design of the study, performed experiments and the statistical analyses, contributed to manuscript revision, and approved the submitted version. JC wrote the first draft of the manuscript. All authors contributed to the article and approved the submitted version.

## Disclaimer

This paper describes objective technical results and analysis. Any subjective views or opinions that might be expressed in the paper do not necessarily represent the views of the U.S. Department of Energy or the United States Government.

## Conflict of Interest

The authors declare that the research was conducted in the absence of any commercial or financial relationships that could be construed as a potential conflict of interest.
