# Supplementary material for: Revisiting the Effects of Xenon on Urate Oxidase and Tissue Plasminogen Activator: No Evidence for Inhibition by Noble Gases
Source: Front Mol Biosci. 2020 Sep 11;7:574477. doi: 10.3389/fmolb.2020.574477 (PMC7516214; doi:10.3389/fmolb.2020.574477)
Supplement: Supplementary file 1 [file Table_1.DOCX]

**SUPPLEMENTARY MATERIAL**

**Revisiting the Effects of Xenon on Urate Oxidase and Tissue Plasminogen Activator: No Evidence for Inhibition by Noble Gases**

**(A)**


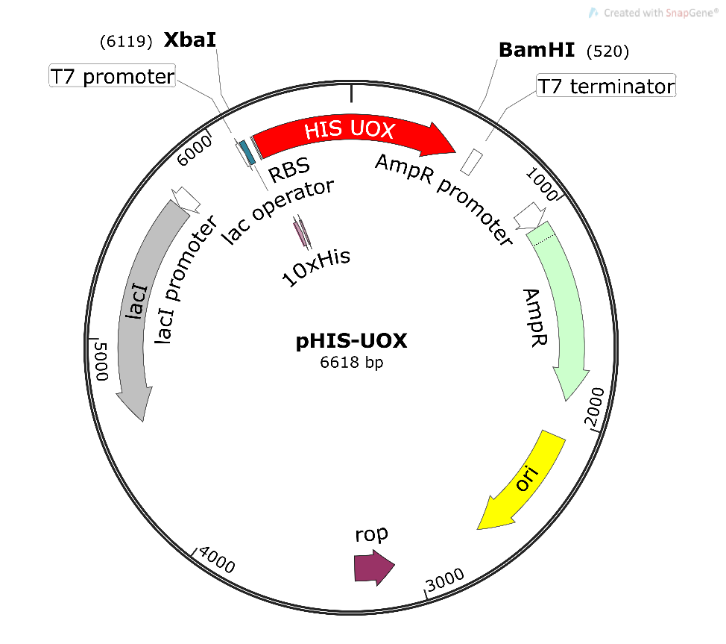


**(B)**

ATGGGCCATCATCATCATCATCATCATCATCATCACAGCAGCGGCCATATCGACGACGACGACAAGCATATGAGCGCGGTGAAAGCGGCGCGTTACGGCAAGGACAACGTGCGTGTTTATAAGGTTCACAAAGATGAAAAGACCGGTGTGCAGACCGTTTACGAGATGACCGTGTGCGTTCTGCTGGAGGGCGAAATCGAGACCAGCTATACCAAGGCGGACAACAGCGTGATCGTTGCGACCGATAGCATTAAAAACACCATCTACATTACCGCGAAGCAAAACCCGGTGACCCCGCCGGAACTGTTCGGTAGCATCCTGGGCACCCACTTTATTGAGAAATATAACCACATCCACGCGGCGCACGTGAACATTGTTTGCCACCGTTGGACCCGTATGGACATCGATGGTAAACCGCACCCGCACAGCTTCATTCGTGACAGCGAGGAAAAGCGTAACGTGCAGGTTGACGTGGTTGAGGGTAAAGGCATCGATATTAAGAGCAGCCTGAGCGGTCTGACCGTGCTGAAGAGCACCAACAGCCAATTCTGGGGCTTTCTGCGTGACGAATACACCACCCTGAAAGAGACCTGGGATCGTATCCTGAGCACCGACGTTGATGCGACCTGGCAGTGGAAAAACTTCAGCGGCCTGCAAGAAGTGCGTAGCCACGTTCCGAAGTTTGATGCGACCTGGGCGACCGCGCGTGAAGTGACCCTGAAAACCTTTGCGGAGGATAACAGCGCGAGCGTTCAGGCGACCATGTATAAGATGGCGGAGCAAATTCTGGCGCGTCAGCAACTGATCGAAACCGTTGAGTACAGCCTGCCGAACAAACACTATTTCGAAATCGACCTGAGCTGGCACAAAGGTCTGCAGAACACCGGCAAGAACGCGGAAGTGTTTGCGCCGCAAAGCGATCCGAACGGTCTGATTAAGTGCACCGTTGGCCGTAGCAGCCTGAAGAGCAAACTGTAA

**(C)**

MGHHHHHHHHHHSSGHIDDDDKHMSAVKAARYGKDNVRVYKVHKDEKTGVQTVYEMTVCVLLEGEIETSYTKADNSVIVATDSIKNTIYITAKQNPVTPPELFGSILGTHFIEKYNHIHAAHVNIVCHRWTRMDIDGKPHPHSFIRDSEEKRNVQVDVVEGKGIDIKSSLSGLTVLKSTNSQFWGFLRDEYTTLKETWDRILSTDVDATWQWKNFSGLQEVRSHVPKFDATWATAREVTLKTFAEDNSASVQATMYKMAEQILARQQLIETVEYSLPNKHYFEIDLSWHKGLQNTGKNAEVFAPQSDPNGLIKCTVGRSSLKSKL*

# Fig. S1. The His-UOX overexpression vector (A), DNA sequence (B) and amino acid sequence (C) of His-UOX are shown.

**
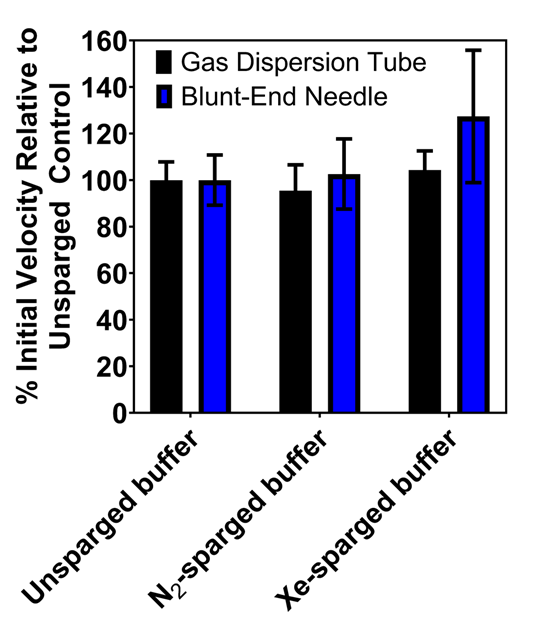
**

# **Fig. S2.** tPA activity measurements with indirect gas delivery, using either a gas dispersion tube or blunt-end needle for sparging the reaction buffer.


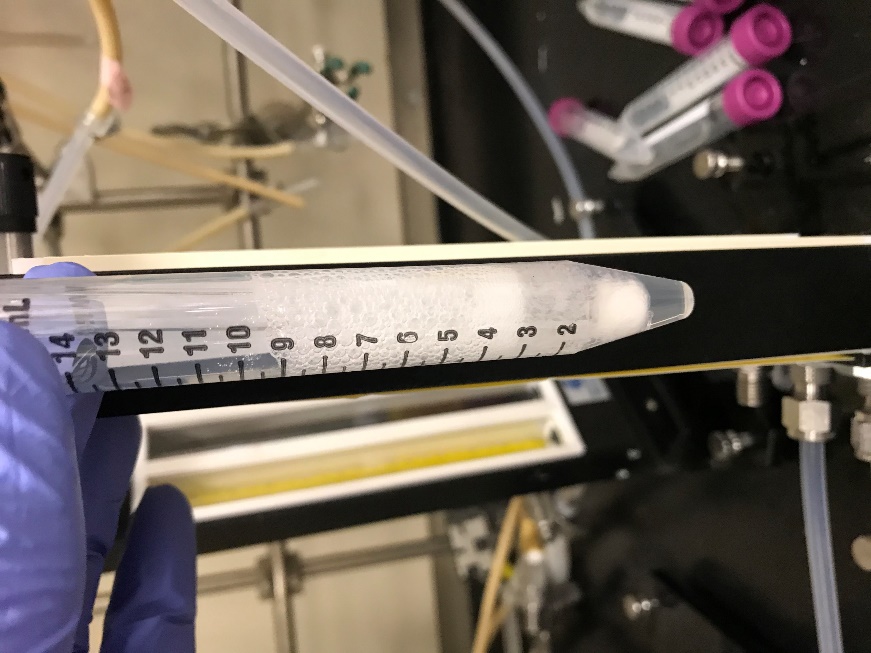


# Fig. S3. Image shows the production of foam (red bracket) when 53 µg/mL His-UOX solution is sparged with Xe/O_2_ or N2/O2 using a gas dispersion tube.


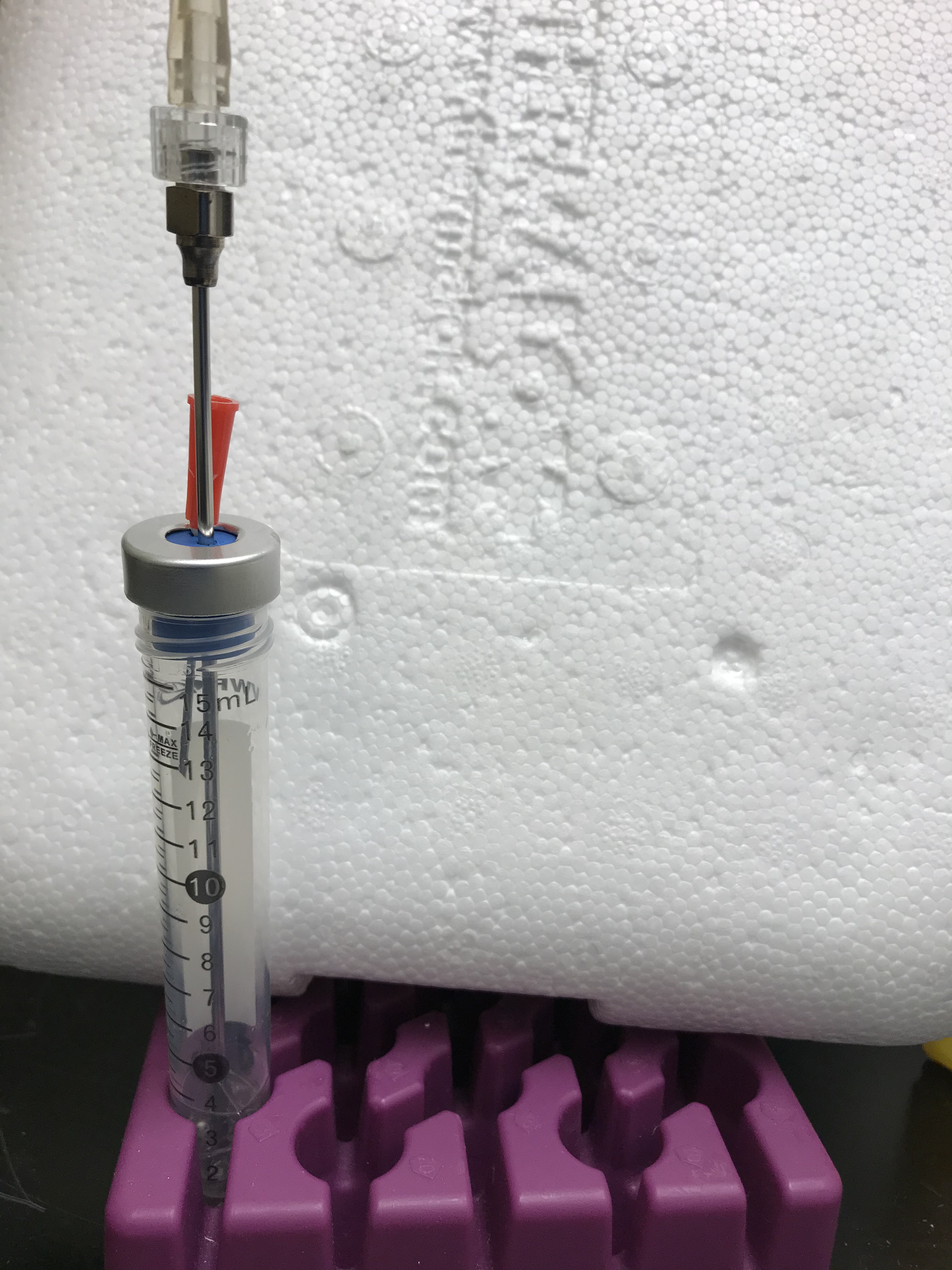

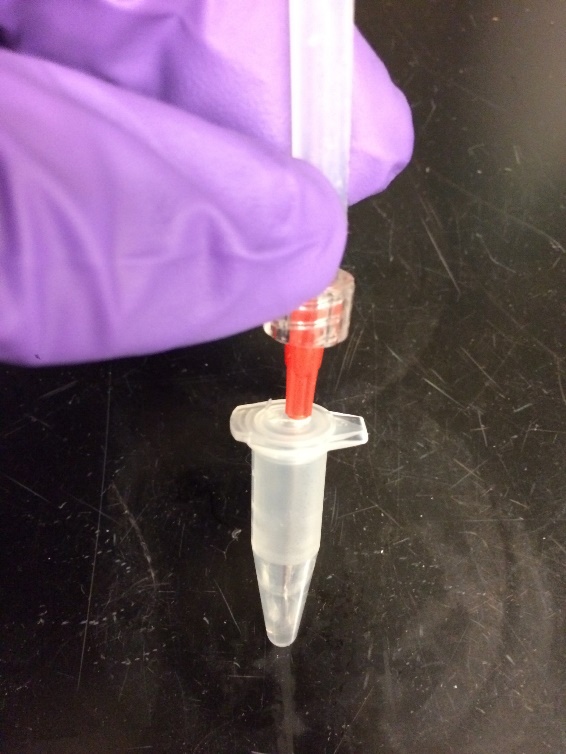


**A**

**B**

# Fig. S4. Image shows alternate methods for delivering gases into reaction buffer or His-UOX solutions using a blunt-end needle (A) 15 mL vented conical tube and (B) vented microcentrifuge tube.
